# The Potential of Atractylodin-Loaded PLGA Nanoparticles as Chemotherapeutic for Cholangiocarcinoma

**DOI:** 10.31557/APJCP.2020.21.4.935

**Published:** 2020-04

**Authors:** Nadda Muhamad, Tullayakorn Plengsuriyakarn, Chuda Chittasupho, Kesara Na-Bangchang

**Affiliations:** 1 *Chulabhorn International College of Medicine, *; 2 *Center of Excellence in Pharmacology and Molecular Biology of Malaria and Cholangiocarcinoma, Chulabhorn International College of Medicine, Thammasat University, Pathum Thani, *; 3 *Department of Pharmaceutical Sciences, Faculty of Pharmacy, Chiang Mai University, Chiang Mai, Thailand. *

**Keywords:** Atractylodin, cholangiocarcinoma, poly (lactic-co-glycolic acid) (PLGA), polymeric nanoparticles

## Abstract

**Backgrounds:**

The anti-cholangiocarcinoma (CCA) activity of atractylodin isolated from *Atractylodes lacea* (Thunb.) DC. has previously been demonstrated both in vitro and in vivo. However, the compound is insoluble in water and must be dissolved in organic solvent which might be harmful to human body. The aim of the study was to develop atractylodin-loaded poly (lactic-co-glycolic acid) (PLGA) nanoparticles (NPs) (ALNPs) and to investigate its cytotoxic activity against CCA.

**Methods:**

The ALNPs were prepared using PLGA MW 12,000 and 48,000 by solvent displacement methods. Particle size, polydispersity index (PDI), zeta potential, encapsulation efficiency (%EE) and loading efficiency (%LE) as well as drug releasing profile of ALNPs were characterized. The selected ALNPs formulation was then investigated cytotoxic activity against CCA cell lines, CL-6 and HuCC-T1.

**Results:**

The ALNPs preparation was achieved using PLGA MW 12,000 (ALNPs-1) with mean (±SD) values of particle diameter, PDI and zeta potential of 158.13±0.21 nm, 0.076±0.003, and (-) 23.80± (-) 0.75 mV, respectively. The transmission electron microscopy (TEM) showed spherical morphology of NPs. The %EE and %LE were 50.16±1.77% and 2.22±0.08%, respectively. The release of atractylodin from ALNPs-1 in PBS was up to 88% in 72 h. The potency of ALNPs-1 cytotoxic activity including selectivity against CCA cell line, CL-6, were about twice of the unformulated atractylodin after 24 h of exposure (IC_50_: 29.28 vs 56.36 µg/mL, selectivity index 2.99 vs 1.50).

**Conclusion:**

ALNPs were successfully prepared by solvent displacement method using PLGA MW 12,000 (ALNPs-1) with suitable pharmaceutical properties and cytotoxic activity against CCA. However, nano-formulation with improved pharmaceutical properties (higher %EE and %LE) and cytotoxic activity (improved selectivity to CCA) should be further developed for potential used as drug delivery systems for the treatment of CCA.

## Introduction

Cholangiocarcinoma (CCA) or bile duct cancer arises from the epithelial cells of the intrahepatic or extrahepatic bile ducts. This cancer is the leading cause of death worldwide. The estimated number of deaths in the United State in 2018 is 30,200 cases (Siegel et al., 2018). The highest incidence of CCA (85 cases per 100,000 population per year) is reported from the northeastern region of Thailand (Bridgewater et al., 2014). Standard chemotherapeutic regimens for the treatment of CCA are 5-fluorouracil (5-FU)-based and gemcitabine-based combination therapies with cytotoxic drugs or targeted therapy (Khan et al., 2012; Kuhlmann, 2012; Ramirez-Merino et al., 2013). Nevertheless, their clinical efficacy remains unsatisfactory (Park et al., 2015).

Several traditional medicines and their derivative natural compounds are currently increasingly recognized as potential complementary treatments for cancers. These include baicalin, curcumin, and *Atractylodes lancea* (*Thunb.*) DC. (*A. lancea*) (Mukerjee and Vishwanatha, 2009; Na-Bangchang et al., 2017; Dou et al., 2018). Promising anti-CCA activities of *A. lancea*, a traditional Chinese medicine, and isolated compounds have been well demonstrated both in vitro and in vivo (Plengsuriyakarn et al., 2012; Plengsuriyakarn et al., 2015; Na-Bangchang et al., 2017). Phase I and II clinical trials to evaluate safety, efficacy, and pharmacokinetics of the oral pharmaceutical formulation of standardized extract of *A. lancea* are underway. Atractylodin is one of the bioactive compounds isolated from the rhizomes of *A. lancea*. Similarly to several other natural compounds, atractylodin is insoluble in water but soluble in organic solvents such as ethyl acetate, dimethyl sulfoxide (DMSO), and acetone (ChemFaces, 2018). This physicochemical property would be expected to limit drug absorption and bioavailability. The aim of the present study was to develop the poly (lactic-co-glycolic acid) (PLGA) nanoparticles (NPs) delivering atractylodin formulation with optimal pharmaceutical and pharmacological properties for the treatment of CCA. The nano-formulation could help to enhance water solubility and thus, improve efficacy of atractylodin in CCA. The advantages of PLGA copolymer over other nanoparticle formulations include its potential to deliver both hydrophobic and hydrophilic drugs and applicability of both oral and parenteral administration (Martin-Banderas et al., 2012; Rafiei and Haddadi, 2017).

## Materials and Methods

*Materials*

Atractylodin and 5-fluorouracil (5-FU) were purchased from WAKO, Osaka, Japan. PLGA 50:50, Resomer^® ^(RG502, MW 12,000, inherent viscosity (IV) 0.24 dL/g), Resomer^®^ (RG504, MW 48,000; IV 0.5 dL/g), D-mannitol, and [3-(4,5-Dimethylthiazol-2-yl)-2,5diphenyltetrazolium bromide] (MTT) reagent were purchased from Sigma-Aldrich, MO, USA. Acetone and dimethyl sulfoxide (DMSO) were purchased from Fisher scientific, Co. LLC, MA, USA. Kolliphor^®^ P 407 (poloxamer 407) was kindly provided by BASF Thailand. The dialysis membrane MWCO 50,000 Da was purchased from Spectrum Laboratory Products Inc., Rancho Dominguez, CA, USA. The CCA cell line CL-6 was kindly provided by Associate Professor Dr. Adisak Wongkajornsilp, Faculty of Medicine, Siriraj Hospital, Mahidol University, Thailand. The CCA cell line HuCC-T1 and human normal fibroblast cell line OUMS-36T-1F were purchased from Japanese Collection of Research Bioresources (JCRB) Cell Bank, Osaka, Japan. RPMI (Roswell Park Memorial Institute) 1640 medium (RPMI) and Dulbecco’s Modified Eagle Medium (DMEM), fetal bovine serum (FBS), and antibiotic-antimycotic solution (100 IU/mL) were purchased from Gibco Co. Ltd., NY, USA.

*Preparation of atractylodin-loaded PLGA NPs*

Two formulations of atractylodin-loaded PLGA NPs (ALNPs-1 and ALNPs-2) and their corresponding blanks PLGA NPs (blank NPs-1 and blank NPs-2) were prepared by solvent displacement method with modification (Martin-Banderas et al., 2012). Atractylodin (1 mg) and PLGA (22.5 mg each of 12,000 or 48,000 MW for ALNPs-1 and ALNPs-2, respectively) were dissolved in acetone (1.5 ml) and thoroughly mixed. The mixture was slowly added into 1% poloxamer 407 (15 mL) using syringe pump (KD Scientific, USA) under magnetic stirring (405 rpm). The excess surfactant was removed by dialysis (MWCO 50,000 Da) against 0.2% D-mannitol solution for 1.5 h. Both ALNPs formulations were kept in the form of suspension at 4°C for further use. Blank NPs were prepared by the same method but without the addition of atractylodin. The formulations of ALNPs and blank NPs are shown in Table 1.

*Characterization of atractylodin-loaded PLGA NPs*

Size, polydispersity index (PDI), and zeta potential values of both ALNPs formulations and their corresponding blanks were investigated by dynamic light scattering (DLS) technique. ALNPs or blank NPs suspension (100 µL of 1.4 mg/mL) was diluted with 900 µL of ultrapure water. The size, PDI, and zeta potential were determined using Zetasizer Nano ZS at the scattering angle of 173° under the temperature of 25°C (Zetasizer Nanoseries, Malvern, UK). The morphology of the selected ALNPs and blank NPs were observed under transmission electron microscope (TEM; JEM-2100 Plus, JEOL, Tokyo, Japan) with an accelerating voltage of 120 kV. The photographs were taken at appropriate magnification. 

*Determination of drug encapsulation and drug loading efficiency*

To determine encapsulation efficiency (%EE) and loading efficiency (%LE), freshly prepared ALNPs suspension (500 µL) was centrifuged (13,000 rpm, 4 °C) for 15 min. The supernatant was discarded and DMSO (500 µL) was added to dissolve drug and polymer. Absorbance of the solution was determined spectrophotometrically using UV Absorbance Reader (Spectramax microplate reader, Molecular Devices, CA, USA) at the wavelength of 340 nm. The amount of atractylodin in the solution was estimated from the calibration curve. The %EE and %LE were calculated from Equation (1) and (2):

$\mathrm{Encapsulation efficency}\left(\mathrm{\%EE}\right)=\frac{\mathrm{Amount of drug loaded in NPs}}{\mathrm{Amount of drug added}}\times 100$

(1)

(2) $$\mathrm{Loading efficency}\left(\mathrm{\%LE}\right)=\frac{\mathrm{Amount of drug in Nps}}{\mathrm{Amount of NPs}}\times 100$$

*Determination of in vitro drug release*

To investigate drug release from both formulations of ALNPs, newly prepared ALNPs suspension (400 µL of 1.4 mg/mL) was suspended in 400 µL of PBS (pH 7.4), serum-free RPMI medium, or serum-free DMEM medium and incubated at 37°C for 20, 40 min, 1.5, 4, 24, 48, and 72 h. The samples were collected at specified time points and supernatant was removed through centrifugation (13,000 rpm, 4°C) for 15 min. DMSO (400 µL) were added to dissolve atractylodin and polymer. The amount of atractylodin released from each ALNPs formulation was determined spectrophotometrically using UV Absorbance Reader (Spectramax Microplate Reader, Molecular Devices, CA, USA) at the wavelength of 340 nm. Cumulative release (%) of atractylodin was calculated according to the Equation (3):

$\mathrm{Cumulative atractylodin release}\left(\%\right)=\frac{\mathrm{DL}-\mathrm{DR}}{\mathrm{DL}}\times 100$

(3)

Where DL: Amount of drug loaded in NPs

DR: Amount of drug remained in NPs

*Cytotoxicity assay*

Human cholangiocarcinoma (CCA) cell lines, CL-6 and HuCC-T1, and normal fibroblast cell line, OUMS-36T-1F, were used for cytotoxicity assay. CL-6 and HuCC-T1 cell lines were cultured in RPMI medium and OUMS-36T-1F cell line was cultured in DMEM medium. Both culture media were prepared as complete medium supplemented with 10% (v/v) FBS and 1% antibiotic-antimycotic solution (100 IU/mL). All cell cultures were maintained at 37°C under 5% CO_2_ atmosphere and 95% humidity (HERRACELL 150i, Thermo scientific, MA, USA).

For cytotoxicity assay, the CCA and normal cell lines were exposed to various concentrations of ALNPs-1 (47-6,000 µg/mL, 100 µL each), blank NPs-1 (47-6,000 µg/mL, 100 µL each), atractylodin (1-125 µg/mL), 5-FU (positive control, 1-125 µg/mL), or serum-free medium (negative control) in a 96-well plate. Briefly, 8,000 cells (in 100 µL complete medium) were seeded into each well and pre-incubated at 37°C under 5% CO_2_ for 24 h. Viability of each cell following exposure to ALNPs-1, blank NPs-1, atractylodin, and 5-FU was determined by MTT assay at 24, 48, and 72 h of exposure (Chittasupho et al., 2017). Absorbance of cell suspension was measured at 550 nm using UV Absorbance Reader (Vario skan flash, Thermo Fisher scientific, MA, USA). Cell viability was calculated using the Equation (4):

$\mathrm{Cell viability}\left(\%\right)=\frac{\mathrm{Absorbance of cell treated with sample}}{\mathrm{absorbance of negative control cells}}\times 100$

(4)

IC_50_ (concentration that inhibits cell growth by 50%) of each compound in each cell line was determined using Graphpad Prism version 7.04 (GraphPad Software, CA, USA). Selectivity index (SI) was determined from the ratio of IC_50_ of ALNPs-1, atractylodin, or 5-FU in normal cell and CCA cell.

*Data analysis*

Quantitative data are presented as mean ± SD or range values of three replications. Inferential statistics for comparison of difference between the groups was not performed due to small sample size.

## Results

*Characterization of atractylodin-loaded PLGA NPs*

**Figure 1:**
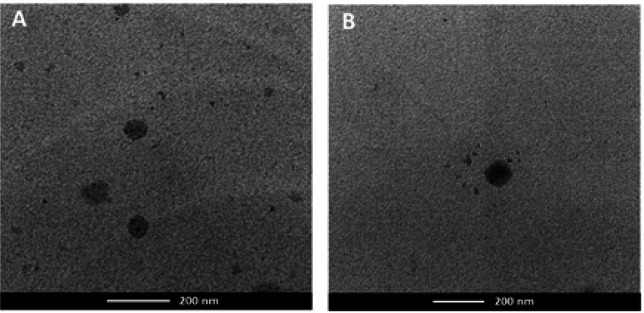
TEM Images of (A) ALNPs-1 and (B) Blank NPs-1

The particle size, PDI, and zeta potential of the prepared ALNPs and blank NPs determined by DLS technique are shown in Table 2. The particle diameter, PDI and zeta potential values of all NP formulations were in the ranges of 150-160 nm, 0.068-0.089 and (-) 29-(-) 23 mV, respectively. The TEM micrographs showed that the size of ALNPs-1 and blank NPs-1 were about 100 nm in diameter. The NPs were well-formed with the spherical shape without aggregation. The morphology ALNPs-1 and blank NPs-1 were shown in Figure 1.

*Drug encapsulation and drug loading efficiency*

Results showed satisfactory encapsulation efficiency (%EE) and loading efficiency (% LE) of both ALNPs formulations (ALNPs-1 and ALNPs-2) (Table 2). The %EE and %LE of ALNPs-1 were about 8-10% higher than ALNPs-2.

*In vitro drug release*

**Figure 2:**
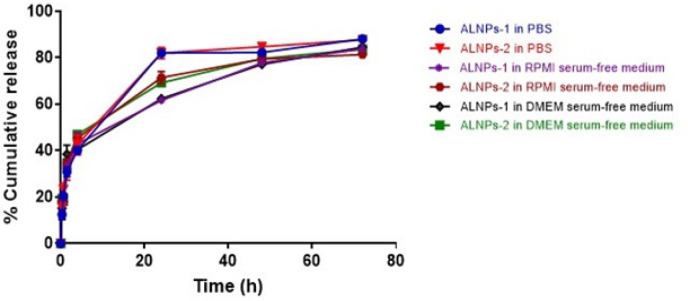
Drug Release Profiles of ALNPs-1 and ALNPs-2 in Various Media. Data are presented as mean ± SD of three replications

Biphasic phases of compound release from both formulations were observed with initial burst release followed by sustained release. The releasing profile of ALNPs-1 and ALNPs-2 in all media are shown in Figure 2. The amounts of atractylodin released from both ALNPs formulations in all media were comparable during both phases. During the initial burst phase (0-4 hours), cumulative release of atractylodin from ALNPs-1 and ALNPs-2 (range values of mean ± SD) in all media were 40.14±0.31-42.87±4.74% and 42.66±4.04-46.92±0.76%, respectively. At 24 hours, the cumulative release were 61.67±0.99-82.06±1.68% and 69.16±0.70-82.03±2.39%, respectively. At 48 hours, the cumulative release were 77.17±1.06-82.30±0.28% and 79.49±1.81- 84.77±0.34%, respectively. At 72 hours, the cumulative release were 83.86±1.01-88.17±0.78% and 81.37±0.58-87.70±0.47%, respectively. Based on the relatively higher %EE however, ALNPs-1 formulation was selected for further study for cytotoxic activity.

*Cytotoxic activity*

**Figure 3:**
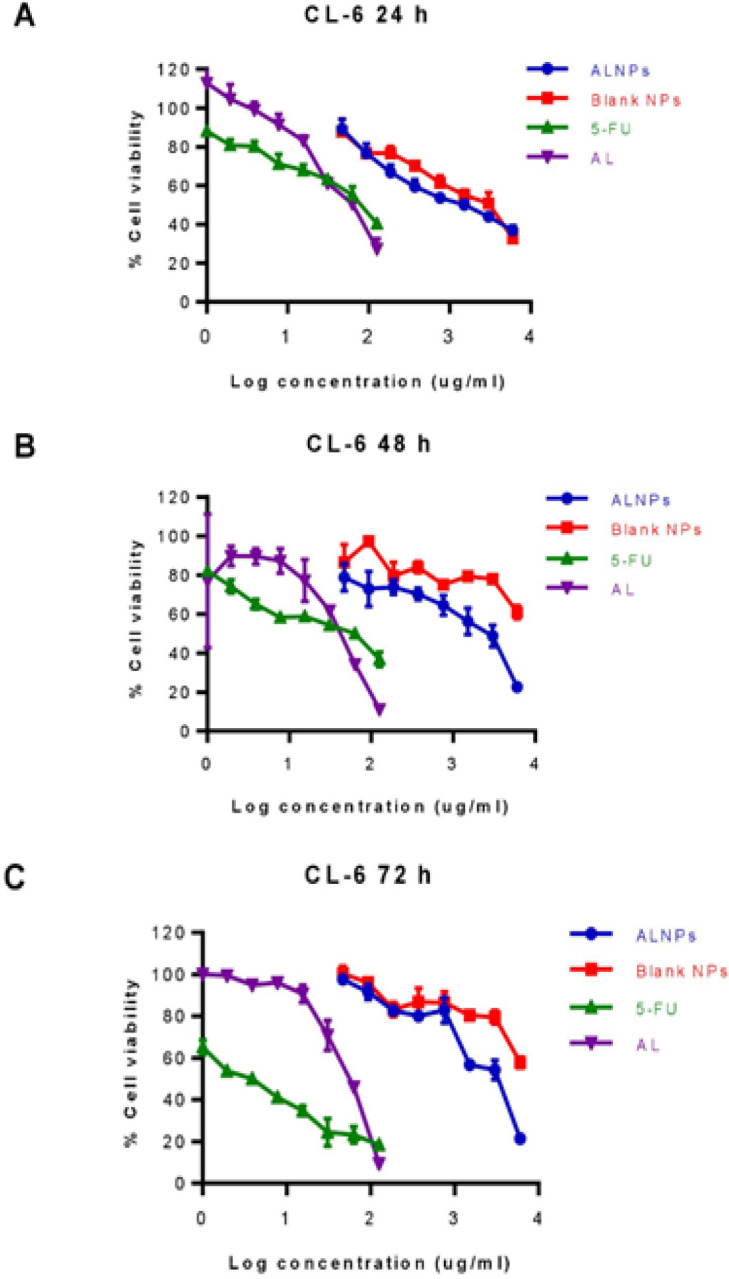
Viability (%) of CL-6 Cells Following Exposure to ALNPs-1, Blank NPs-1, Atractylodin, and 5-FU (Positive Control) for (A) 24, (B) 48, and (C) 72 h. Data are presented as mean ± SD of three replications

**Figure 4:**
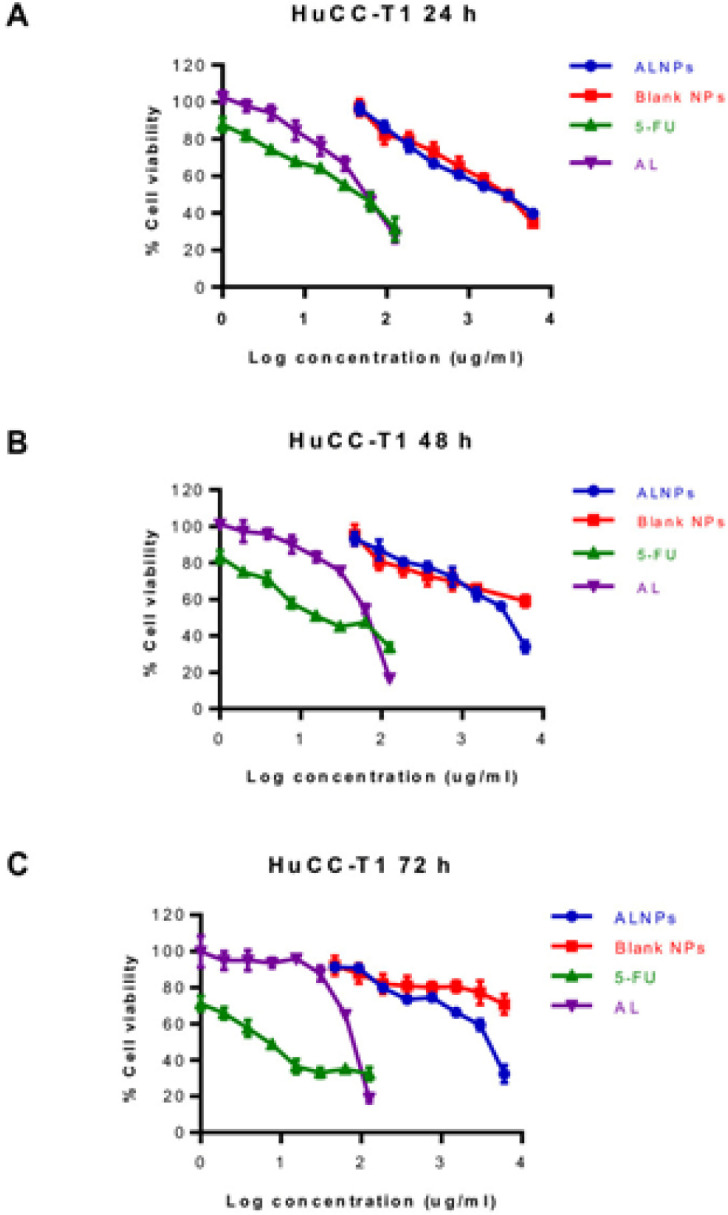
Viability (%) of HuCC-T1 Cells Following Exposure to ALNPs-1, Blank NPs-1, (AL) and 5-FU (Positive Control) for (A) 24, (B) 48, and (C) 72 h. Data are presented as mean ± SD of three replications

**Figure 5:**
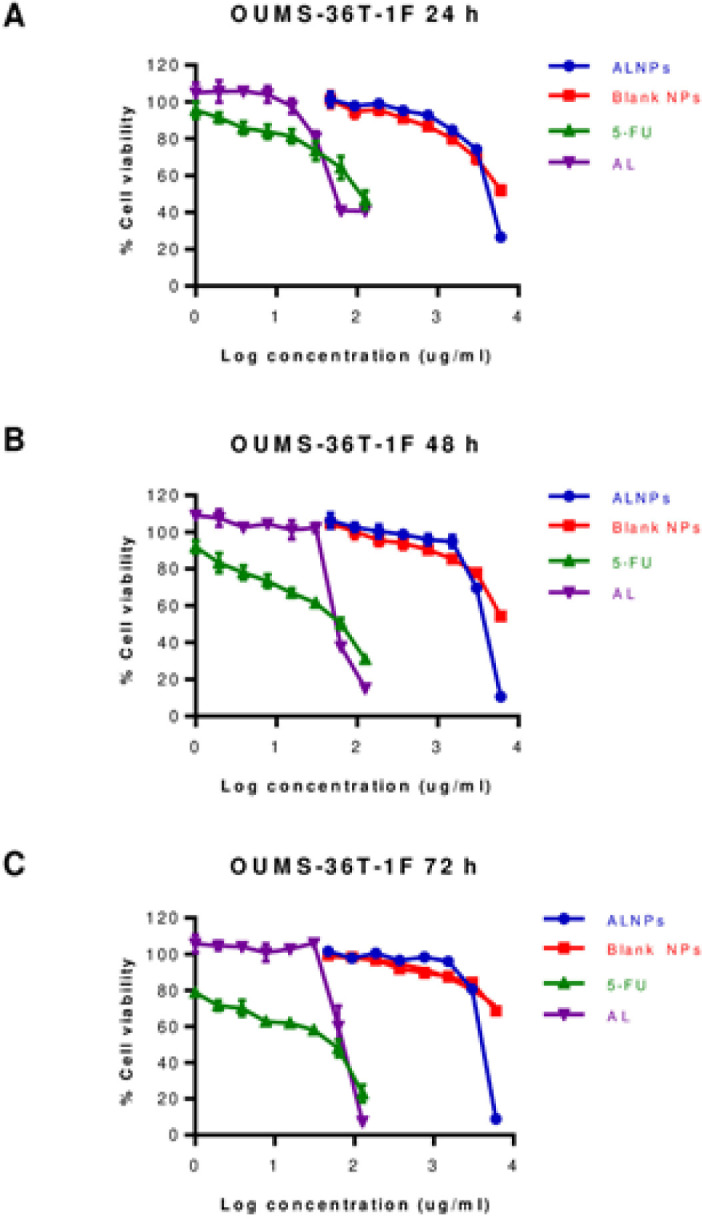
Viability (%) of OUMS-36T-1F Cells Following Exposure to ALNPs-1, Blank NPs-1, (AL), and 5-FU (Positive Control) for (A) 24, (B) 48, and (C) 72 h. Data are presented as mean ± SD of three replications

Cell viability of CL-6 after exposure to the highest concentration of atractylodin (125 µg/mL) and ALNPs-1 (6,000 µg/mL) at 24 hours of exposure were 24.38-33.56% and 34.92-39.55%, respectively. The corresponding values for 48 and 72 hours exposure were 9.71-13.89% vs 21.16-24.48% and 9.12-10.27% vs 20.01-22.21%, respectively (Figure 3). Cell viability of HuCC-T1 after exposure to the highest concentration of atractylodin and ALNPs- 1 at 24 hours of exposure were 23.72-30.39% and 38.36-41.17%, respectively. The corresponding values for 48 and 72 hours exposure were 16.18-17.18% vs 31.02-37.36% and 16.43-20.57% vs 26.82-35.28%, respectively (Figure 4). Cell viability of OUMS-36T-1F after exposure to the highest concentration of atractylodin and ALNPs-1 at 24 hours of exposure were 39.61-41.50% and 25.67-28.42%, respectively. The corresponding values for 48 and 72 hours exposure were 14.88-15.37% vs 8.94-13.02% and 6.61-7.92% vs 7.71-10.97%, respectively (Figure 5). The IC_50_ values of atractylodin, ALNPs-1 and blank NPs-1, and 5-FU for the CL-6, HuCC-T1, and OUMS-36T-1F cell lines after 24, 48, and 72 hours of exposure are presented in Table 3.

The selectivity index (SI) of atractylodin, ALNPs-1, and 5-FU for CL-6 at 24, 48, and 72 hours were 1.50 vs 2.99 vs 1.71, 1.54 vs 2.18 vs 1.28, and 1.33 vs 1.62 vs 8.90, respectively. The corresponding SI of atractylodin, ALNPs-1 and 5-FU for HuCC-T1 at 24, 48, and 72 hours were 1.57 vs 1.83 vs 3.41, 0.97 vs 1.16 vs 1.92, and 0.91 vs 1.15 vs 3.75, respectively.

**Table 1:** Formulations of ALNPs and Blank NPs

Formulation	Atractylodin (mg)	Acetone (mL)	12,000 MW PLGA (mg)	48,000 MW PLGA (mg)	1% Poloxamer 407 (mL)
ALNPs-1	1	1.5	22.5	-	15
ALNPs-2	1	1.5	-	22.5	15
Blank NPs-1	-	1.5	22.5	-	15
Blank NPs-2	-	1.5	-	22.5	15

**Table 2:** Particle Size, Polydispersity Index (PDI), Zeta Potential Values, Encapsulation Efficiency, and Loading Efficiency of ALNPs and Blank NPs. Data are presented as mean ± SD values of three replications

Formulation	Size (nm)	PDI	Zeta potential value (mV)	Encapsulation efficiency (%EE)	Loading efficiency (%LE)
ALNPs-1	158.13±0.21	0.076±0.003	-23.80±0.75	50.16±1.77	2.22±0.08
ALNPs-2	161.27±1.87	0.068±0.015	-28.83±0.35	46.06±1.59	2.05±0.07
Blank NPs-1	159.43±0.68	0.089±0.023	-24.60±1.21	-	-
Blank NPs-2	156.10±1.54	0.095±0.023	-29.13±0.32	-	-

**Table 3:** Cytotoxic activities of Atractylodin, ALNPs-1, Blank NPs-1 and 5-Fluorouracil (5-FU) for the CL-6, HuCC-T1 and OUMS-36T-1F Cell Lines after 24, 48, and 72 h of Exposure. Data are presented as mean ± SD values of IC_50_ from three replications

Sample	IC_50 _(µg/mL)								
	CL-6			HuCC-T1			OUMS-36T-1F		
	24 h	48 h	72 h	24 h	48 h	72 h	24 h	48 h	72 h
Atractylodin	56.36	37.66	52.02	53.66	59.74	76.15	84.33	58.16	69.03
ALNPs-1 (eq. to Atractylodin)	1,412 (29.28)	1,694 (35.06)	2,447 (50.74)	2,299 (47.68)	3,189 (66.09)	3,443 (71.30)	4,185 (87.41)	3,662 (76.55)	3,930 (82.10)
Blank NPs-1	2,112	>6,000	>6,000	2,393	>6,000	>6,000	>6,000	>6,000	>6,000
5-FU	77.43	38.84	3.46	38.87	25.95	8.2	132.7	49.83	30.78

## Discussion

In the present study, two atractylodin-loaded PLGA NPs (ALNPs) formulations were prepared by solvent displacement method to enhance water solubility of atractylodin using PLGA MW 12,000 (ALNPs-1) and 48,000 (ALNPs-2). The particle diameters were in the range of 100-200 nm which are suitable to be used as a drug delivery system for cancer treatment to facilitate the enhance permeability and retention (EPR) effect (Huang and Zhang, 2018). The effect of MW of PLGA on the size of ALNPs was not observed in this study. In a previous study, smaller NPs size was observed with NPs loaded hydrophobic drugs prepared using higher MW PLGA due to longer aliphatic chain (Martin-Banderas et al., 2012). On the other hand, another study reported smaller size of NPs prepared using lower MW PLGA (Surolia et al., 2012). The PDI values of both ALNPs formulations was less than 0.1 indicating monodisperse size distribution (Gossmann et al., 2015). As ALNPs-1 exhibited higher %EE, thus ALNPs-1 and the corresponding blank NPs-1 were selected for morphology investigation. TEM showed no difference between the morphology of drug-loaded NPs and blank NPs. This confirmed that the drug loaded in the NPs did not affect the size and shape of the NPs. Both ALNPs and blank NPs were spherical shape without an aggregation. The size of NPs examined by TEM was smaller than that observed from DLS. The DLS technique measures hydrodynamic diameter including the core and the charge layer surrounded on the particle, while the TEM measures the size of particles at the dry state and in neutral environment resulted in smaller particle size (Gaumet et al., 2008; Swider et al., 2018). For the surface charge, both ALNPs formulations provided zeta potential lower than (-) 20 mV which would be expected to promote the stability of NPs (Honary and Zahir, 2013). High MW surfactants such as poloxamer 407 used to prepare NPs have been shown to provide zeta potential (-)20 mV or much lower with sufficient stability for NP suspensions (Honary and Zahir, 2013). The ALNPs-1 was shown to provide higher encapsulation efficiency (%EE) and loading efficiency (%LE) compared with ALNPs-2. This might be due to the solid-state solubility of atractylodin in the polymer matrix during dispersed state (Panyam et al., 2004). The amount of hydrophobic drug-entrapped into hydrophobic polymer due to solid-state drug-polymer has been shown to be increased with the increasing lactide content in copolymer and with the decreasing in MW of polymer (Panyam et al., 2004). Atractylodin encapsulated in ALNPs at the concentration up to 500 mg/L was freely dispersible in water, while the solubility of free atractylodin in water was only 7.541 mg/L ( TGSC Information System, 2019). This indicated that ALNPs facilitated water dispersibility of atractylodin as a result of the adsorption of poloxamer on hydrophobic surface of PLGA NPs and the expression of hydrophilic part (polyethyleneoxide) to the aqueous solution (Santander-Ortega et al., 2006; Pradhan et al., 2013). 

Drug releasing profiles of both ALNPs formulations were shown to be similar with biphasic phases in all media. This result confirms the release of atractylodin from ALNPs to produce cytotoxic effect. The burst release from NPs is explained by diffusion of the adsorbed atractylodin on the surface of NPs through the polymer matrix as well as by the large surface to volume ratio of the NPs. The slow release phase is explained by drug diffusion and polymer degradation (Chittasupho et al., 2009; Mukerjee and Vishwanatha, 2009). Previous studies demonstrated higher rate of drug release from low MW compared with high MW PLGA NPs since PLGA with high MW contains higher content of the hydrophobic part lactic acid which results in lower amount of drug release from the NPs (Mittal et al., 2007; Surolia et al., 2012). The drug release from ALNPs-1 in PBS (pH 7.4) after 24 hours of incubation was significantly higher than that in DMEM-serum free medium (pH 8.85) and RPMI-serum free medium (pH 9.06). The higher drug release rate in PBS was due to the lower pH of PBS which facilitated hydrolysis of PLGA copolymer and resulted in higher amount of compound release from the nanoparticles compared to drug release into DMEM-serum free medium and RPMI-serum free medium (Ham et al., 2008; Khanal et al., 2016; Chai et al., 2017). However, after 48 and 72 hours, the amount of atractylodin released was comparable in all media. This might be due to the saturation of atractylodin in each media that led to delay of compound release from NPs. 

Cytotoxic activity of ALNPs-1 against the two CCA cell lines (CL-6 and HuCC-T1) was investigated using MTT assay following the optimized conditions. Results suggested that ALNPs-1 was successfully prepared as indicated by its relatively potent cytotoxic activity against both CCA cell lines. However, the potency of cytotoxic activity of ALNPs-1 against both CCA cell lines following 48 and 72 hours of exposure was about 2-14 folds lower than 5-FU. The cytotoxic activity of atractylodin and ALNPs-1 appeared to be concentration-dependent. Atractylodin exhibited cytotoxicity against both HuCC-T1 and CL-6 at the concentration of 31.25 µg/mL (%cell viability less than 70%) which was lower than that of ALNPs-1 (%cell viability less than 70% at 7.81 µg/mL) when exposing to both cells for 24 hours. After removing NPs from the medium, the cytotoxicity of both atractylodin and ALNPs-1 for both cancer cells were decreased due to recovery of the cells resulting in higher IC_50_ values at 48 and 72 hours. Even though atractylodin at high concentrations (62.5-125 µg/mL) produced cytotoxic effect against normal cells (OUMS-36-1F), the IC_50_ of AL was higher than that of cancer cells. The cytotoxicity of blank NPs was observed at high concentration due to the unsuitable environment for cell growth (cloudy of NP suspension in culture medium). However, the cells recovered after replacement with complete medium. In the previous study, the recovery of the cells was observed after 5 and 7 days of treatment with blank PLGA microsphere (Azouz et al., 2008). 

The ALNPs were successfully prepared by solvent displacement method using PLGA MW 12,000 (ALNPs-1) with suitable pharmaceutical properties and cytotoxic activity against CCA. However, nano-formulation with improved pharmaceutical properties (higher %EE and %LE) and cytotoxic activity (improved selectivity to CCA) should be further developed for potential used as drug delivery systems for the treatment of CCA.
